# The sweet tooth of infancy: Is sweetness exposure related to sweetness liking in infants up to 12 months of age?

**DOI:** 10.1017/S0007114522002628

**Published:** 2023-04-28

**Authors:** Carina Müller, Claire Chabanet, Gertrude G. Zeinstra, Gerry Jager, Camille Schwartz, Sophie Nicklaus

**Affiliations:** 1 Division of Human Nutrition and Health, Wageningen University and Research, Stippeneng 4, 6703 HD, Wageningen, The Netherlands; 2 Centre des Sciences du Goût et de l’Alimentation, CNRS, INRAE, Institut Agro, Université Bourgogne Franche-Comté, Dijon, France; 3 Wageningen Food & Biobased Research, Wageningen University & Research, PO 17, 6700 AA, Wageningen, the Netherlands

**Keywords:** Sweetness liking, Sweetness exposure, Children, Longitudinal, Infants, Sweetness preference, Birth cohort, Sugar

## Abstract

Infants become increasingly exposed to sweet-tasting foods in their first year of life. However, it is still unclear whether repeated exposure to sweet taste is linked to infants’ sweetness liking during this period. Making use of data from the OPALINE cohort, this study aimed to examine the link between sweetness exposure and sweetness liking during two important periods in early infant feeding: at the start of complementary feeding (3–6 months) and the transition to the family table (10–12 months). Infants’ sweetness exposure was assessed using 7-d food records which were completed by mothers every month (*n* 312), reporting daily consumption rates of formula/breast milk or complementary food and the type of formula milk and/or complementary foods for each feeding occasion. Infants’ sweetness liking was studied in the laboratory at 3, 6 and 12 months of age by assessing their response to a lactose–water solution and the amount drunk of this solution compared with plain water. Linear regressions and structural equation model assessed associations between exposure to and liking for sweetness at 6 and 12 months. Neither at 6 (*n* 182) nor at 12 months (*n* 197) was sweetness exposure associated with sweetness liking. While sweetness liking at 3 months was unrelated to liking at 6 months, the latter predicted sweetness liking at 12 months. These findings demonstrate no association between sweetness exposure at 3 to 12 months and liking at 6 and 12 months despite a sharp increase in sweetness exposure in that period. However, sweetness liking at 6 and 12 months was positively associated.

Humans have an innate preference for sweet taste^(1–3)^. In childhood, the sweet taste is generally more liked than in adulthood and is preferred over all other tastes in the first 20 months of life^(4,5)^. Despite the universal trait of liking sweetness, there are great inter-individual differences regarding which specific tastes and sweetness levels are liked the most^(6)^. A child’s preferred level of sweetness may ultimately affect his or her food intake and weight, as a greater preference for sweetness has been associated with consumption of high-energy foods and, moreover, with overweight and obesity in 6-to-9-year-old children^(7,8)^. However, it remains difficult to explain how differences in sweetness liking arise^(9)^. A body of research argues that food and taste preferences may be linked to sex^(10)^ or genetic differences^(11)^. Previously, boys were found to like sweet foods more at 8 to 12 years than girls^(10)^, and genotypes at the TAS2R38 taste gene locus were associated with sucrose preference and liking of sweet-tasting foods in 5-to-10-year-old children^(11)^. Further, research suggests that individuals vary in their hedonic liking response to sweetness due to their phenotype^(12)^ and that children move along a continuum between sweet likers and sweet dislikers^(6)^.

In addition to sex and genetic differences, there is an ongoing discussion about whether repeated exposure to sweet taste alters the degree of sweetness liking or not^(13–17)^. Some research suggests that sweetness liking may be maintained or increased through dietary exposure to sweet-tasting foods^(15–17)^. For example, babies who were regularly fed sweetened water during their first months of life liked sweetened water more at 6 months and at 2 years than children who were not regularly fed sweetened water in infancy^(16,17)^. However, this link was not found when sweetness liking was tested with a sweetened fruit-flavoured drink instead of sweetened water at 2 years of age^(17)^. This suggests that the relationship between early sweetness exposure and sweetness liking may depend on the medium used for both exposing to sweetness and measuring sweetness liking. In a recent systematic review, equivocal evidence for the presence and possible direction of a relationship between sweetness exposure and sweetness liking was found^(18)^. On the one hand, a higher intake of added sugars in the first year of life was related to a higher preference for sweetness at the age of 4 to 7 years^(14)^. Further, a higher intake of foods with added sugars during the first year of life was linked to a higher intake at the age of 3 to 7 years^(13)^. On the other hand, neither added sugar consumption nor the consumption frequency of sweet foods was linked to sweetness liking in 7-to-12-year-old and 6-to-9-year-old children, respectively^(8,19)^. Those findings demonstrate that the link between sweetness exposure and liking is more complex than initially indicated by earlier studies, and far from being systematically shown.

In addition to the different study results and conclusions, very limited research aims to shed light on the association between exposure to sweet taste and the degree of sweetness liking in the first 12 months of life^(20)^. During this time, infants go through a major dietary transition – from exclusive milk feeding over complementary feeding to eating what is consumed by the family^(21,22)^. In addition, what infants eat in those early months could influence their eating behaviour in the long term, as diet quality and food preferences in infancy are related to food intake and food preferences through adolescence and early adulthood^(23–27)^. Moreover, research suggests that dietary patterns are already established by the age of 18 months, and those set at 2 years build the root for lifelong eating habits^(21)^. Due to the importance of the first few months for long-term eating behaviour, it is important to examine this period to assess the role of or potential relationship between repeated exposure to sweet-tasting foods and the degree of sweetness liking at this crucial stage of life.

When dietary intake is no longer limited to breast or formula milk, the level of sweetness exposure starts to differ more between children^(28,29)^. Hence, this study was conducted to define the role of dietary exposure to sweetness on sweetness liking during two crucial changes in early infant feeding: at 3 to 6 months (start of complementary feeding) and at 10 to 12 months (transition to the family table). We predicted that higher exposure to sweetness would be associated with higher sweetness liking in those periods. Further, we hypothesised that the link between sweetness exposure and sweetness liking would be stronger at 12 than 6 months, as infants’ sweetness exposure was found to be higher at 10 to 12 compared with 3 to 6 months^(28)^. Acknowledging that sweetness liking may already differ between infants at birth, we also investigated the associations between sweetness liking at 3 months and sweetness liking at 6 and 12 months. We hypothesised that repeated exposure would override innate sweetness preferences so that sweetness liking at 3 months would have a low impact on sweetness liking at 6 and 12 months.

## Methods

### General design

We conducted secondary data analyses using the French birth cohort study ‘Observatoire des Préférences Alimentaires du Nourrisson et de l’Enfant’ (Observatory of Infant and Child Food Preferences), OPALINE in short. Between 2005 and 2009, 312 women were recruited before their last trimester of pregnancy via leaflets and posters at doctors’ and pediatricians’ consulting rooms in maternity hospitals and clinics, as well as pharmacies and day care centres. To be included in the study, parents had to be at least 18 years old and have children in good health after birth. Children’s degree of sweetness liking was assessed in the laboratory at ages 3, 6, 12 and 20 months (based on the expected delivery date, not the actual delivery date). In this paper, only the measurements at 3, 6 and 12 months are included in the analyses as the procedure at 20 months was slightly different^(4)^. During their last trimester of pregnancy, mothers participated in interviews on sociodemographic and health characteristics. After the child’s birth, mothers reported their children’s birth date, sex, gestational age, and birth weight and length. Further, between birth and 1 year, mothers filled out 12 monthly 7-d food records at home in which they indicated the number and timing of each feeding occasion with breast or formula milk. Furthermore, they described the complementary foods they might have given, the food type (home-made food, ready-prepared baby food, ready-prepared adult food, plus the brand if relevant), and texture (e.g. drained, lumpy or thick), and whether they had added any ingredient (e.g. sugar or salt) to the foods. They were not asked to report the amount consumed by the child. Mothers also recorded special occurrences in between two monthly food records, such as the introduction of new foods. Based on the records, foods were grouped into milk, home-made foods, ready-prepared baby foods and ready-prepared adult foods. Detailed explanations of the recording method and overall procedures used in the OPALINE study were explained previously^(4,28,30,31)^. This study was conducted according to the guidelines laid down in the Declaration of Helsinki, and all procedures involving human subjects were approved by the local ethical committee (Comité Consultatif de Protection des Personnes dans la Recherche Biomédicale de Bourgogne-ID RCB 2007-A00808–45). Written informed consent was obtained from both parents prior to the first experimental session. The protocol related to the present analysis was preregistered at https://osf.io/6tz4c. The OPALINE data can be accessed upon request.

### Sweetness exposure

Infants’ exposure to sweetness (SweetExposure) was measured through the diet (based on the food records) as the cumulative frequency of the taste intensities of all consumed foods during each period of recording^(28,32,33)^. For that, all foods were linked to their sweetness intensity through existing databases^(32,33)^. In short, the data sets (one for baby food and one for adult foods) included information for each food item based on the evaluation from trained panellists who tasted and rated the taste intensity on a continuous scale from 0 to 10, inspired by the Spectrum method^(34)^. Following this method, four sweet reference solutions were used and arranged on the scale with increasing intensity to give reference intensities to the panellists to aim for standardised ratings^(28)^. For the adult food database, the panellists conducted home-based measurements where the reference solutions were replaced by reference foods^(34)^. Mixed dishes were rated according to the mean of the ingredients’ tastes. An extensive explanation of the sweetness intensity measurements can be found elsewhere^(28)^. The exposure to sweetness was then calculated for each month and child by the sum of sweetness intensities of food items weighted by consumption frequencies and divided by the number of days for which the food diary products were completed. Based on that, the average daily mean of SweetExposure was calculated for the 3 to 6 and 10 to 12 months periods.

### Sweetness liking

Infants’ degree of sweetness liking (SweetLiking) was estimated at 3, 6 and 12 months by comparing the infant’s acceptance of a sweet solution (0·20 M lactose + water) to water. The solution was chosen because it is similar to the lactose concentration in breast milk and reflects moderate intensity^(31)^. The sweet solution was prepared every 3 d, kept refrigerated at +4° C and used at room temperature during the sweetness liking test. The bottles contained 30 ml of the solution or water at 3 and 6 months and 50 ml at 12 months. Further, mothers reported which nipple shape and material the child was used so that the experience during the laboratory assessment would be similar to infants’ home experience. In the data collection process, liking was also assessed for the other basic tastes (sour, salty, bitter and umami), and the researchers were blind to the order in which these tastes were tested. For this paper, only the degree of sweetness liking is reported.

### Procedure

Children were tested individually in a room designed for infant testing at the Centre Européen des Sciences du Goût (Dijon, France) or the FLAVIC (FLAvour VIsion Consumer Behaviour) joint research unit (Dijon, France). Children sat in a bouncer (3 and 6 months) or a high chair (12 months). Each of them was accompanied by one parent, while one of the four researchers included in the study conducted the test. To ensure that children were in a similar hunger state at the beginning of the test, parents were asked to refrain from giving the child water, milk or food 1 h before the test. On the test day, compliance with this instruction was checked by asking the parent to report the last time the child had consumed something on that day. In videotaped sessions, children were presented with four bottles in the following order: water, sweet solution, sweet solution and water. Each of the bottles was presented for 45 s by gently rubbing the nipple against the child’s mouth, followed by a 15-s break before presenting the next bottle. In case the child did not drink, he/she was offered a toy. When necessary, the parent sat closer to the child, or the child was put on the parent’s lap. The degree of sweetness liking was defined through three scores: the ingestion ratio (IR) and two liking ratios (liking rated by the experimenter and liking rated by the parent). Sweetness liking measures were only recorded if the child drank at least 1·0 g from the sweet solution. Otherwise, it was considered missing.

### Ingestion ratio

The first indicator for the degree of sweetness liking, the IR, was created by measuring the amount the child drank from the sweet solution bottles and the water bottles. For that, the bottles were weighed to the nearest 0·1 g (Sartorius U3600S; Sartorius AG) before and after each solution was presented to the child. Based on the weights, the IR was calculated by first summing the amount drunk from the two sweet solutions. This sum was then divided by the sum of amounts drunk from all four bottles so that each child could be assigned an IR between 0 and 1.

IR = (Grams sweet bottle 1 + Grams sweet bottle 2) / (Grams Sweet Bottle 1 + Grams Sweet Bottle 2 + Grams Water Bottle 1+ Grams Water Bottle 2)

### Liking ratio

For the second and third sweetness liking indicators, the experimenters and parents rated the child’s reaction to each of the four bottles on a five-point Likert scale (1 – strong rejection; 2 – slight rejection; 3 – neutral; 4 – slight acceptance and 5- strong acceptance). The child’s overall behaviour, including facial expressions, was taken into account. As a result, each of the four bottles was assigned a score between 1 and 5. Based on those scores, the experimenter liking ratio (LRE) and parent liking ratio (LRP) were calculated similarly to the IR by summing the scores of the two sweet solutions and dividing them by the sum of all four scores. Again, ratios between 0 and 1 were possible.

LRE/LRP = (Score sweet bottle 1 + Score sweet bottle 2) /(Score Sweet Bottle 1 + Score Sweet Bottle 2 + Score Water Bottle 1 + Score Water Bottle 2)

For each of the three liking ratios, a ratio of 0·5 indicated indifference to the sweet solution relative to water. A ratio of > 0·5 indicated a preference for the sweet solution relative to water, and a ratio of < 0·5 indicated rejection of the sweet solution relative to water.

### Subjects

In this paper, data were included from 312 mothers, leading to a sample of 319 children including seven pairs of twins. Of those, information on the degree of sweetness liking was available for 153 infants at 3 months, 216 at 6 months and 215 at 12 months. Further, of the 319 infants, information on sweetness exposure was available for 251 infants for the 3 to 6 months period and 264 for the 10 to 12 months period.

For SweetLiking, missing data were considered as occurring at random because they were due to children being sick or drinking less than 1·0 g of the sweet solution on the assessment day. Similarly, missing data for SweetExposure were considered as occurring at random because they were due to incomplete food records. As many observations as possible were included for each analysis, resulting in different sample sizes for each analysis.

### Statistical analyses

Statistical analyses were performed using R, version 4.1.1 for Windows. The sweetness liking data were merged across the experimenters and sessions, as previously no difference was found in the judgement of the experimenters^(31)^. Results are expressed as mean values and standard deviations. Inferences about the association between the degree of sweetness liking and sweetness exposure were made based on *P*-values, the size of regression coefficients and CI.

To assess whether SweetLiking at 3 months differed from SweetLiking at 6 and 12 months and whether liking at 6 months differed from liking at 12 months, paired samples *t* tests were conducted. Again, as many observations as possible were used for each *t* test (*n* 137 (difference 3 and 6 months); *n* 164 (difference 6 and 12 months); *n* 122 (difference between 3 and 12 months)). In addition, another paired samples *t* test was conducted to assess whether exposure to sweetness differed at 3 to 6 months compared with 10 to 12 months, including all individuals with complete data on sweetness exposure at both investigated periods (*n* 247).

The relationship between sweetness exposure and liking was tested using linear regressions. To reduce the number of linear regression models, we only used one of the two liking ratios, because both (LRE and LRP) measured the child’s hedonic liking and correlated strongly with each other at each of the time points of assessment (3 months (r(151) = 0·43, *P* < 0·001); 6 months (r(214) = 0·48, *P* < 0·001); and 12 months (r(209) = 0·64, *P* < 0·001)). The LRE was chosen as it was based on the judgement of the same four experimenters, whereas the LRP was based on the judgement of each parent. All linear regression models were conducted twice, with the IR and LRE as the outcome variables. First, the link between the degree of sweetness liking (IR and LRE) at 6 months (12 months respectively) and exposure to sweetness at 3 to 6 months (10–12 months, respectively) was tested with a bivariate linear regression. For that, all individuals with complete data on IR and LRE at 6 months (12 months) and sweetness exposure at 3 to 6 months (10–12 months) were included in the analyses. Next, multiple linear regressions were conducted by adding confounders to the bivariate models. Based on the directed acyclic graph method, the duration of exclusive breast-feeding and the mother’s level of education were selected as confounders for inclusion in the regression analyses^(4,5,28,35,36)^. Next, an interaction term between the child’s sex and sweetness exposure, as well as the main effect of sex, were introduced but removed again from the final models due to non-significance. Because missing values were judged to be at random, the bivariate analyses were performed a second time for complete cases only to assess the robustness of the results. Only individuals with available data for all four variables (SweetLiking at 6 months, SweetLiking at 12 months, SweetExposure at 3 to 6 months and SweetExposure at 10 to 12 months) were included in the complete case models.

Finally, to get a global picture of the relationships between exposure and liking, all available data regarding SweetLiking at 3, 6 and 12 months and SweetExposure at 3 to 6 and 10 to 12 months were gathered in a structural equation model (supplementary material Fig. S1, *n* 319). Compared with the linear regressions, the structural equation model allows for consideration of all (in)dependent variables in the same model and therefore to test relationships simultaneously^(37)^. Structural models were estimated using the R package Lavaan 0.6–7, by full information maximum likelihood to address missing values^(38)^. All three SweetLiking variables (IR, LRE and LRP) were used for a more precise estimation of liking at each age. The first step was to check whether the construct measurement (confirmatory factor analysis, CFA) was satisfactory concerning the latent variables (SweetLiking at 3, 6 and 12 months) as measured by the three indicators (observed variables IR, LRE and LRP). More precisely, the CFA model was built, and fit criteria [CFI (0.985), TLI (0.978) and RMSEA (0.034)] were used to evaluate the quality of the measurement model and convergent validity. It was checked that the estimation showed high loadings and low correlations between constructs. Next, the structural model was built. SweetLiking at 3 months of age was included to account for individual differences in sweetness liking that may occur during the milk feeding period before differences in sweetness exposure begin to increase. It was assessed whether SweetLiking at 3 months predicts SweetLiking at 6 months, whether SweetLiking at 6 months predicts SweetLiking at 12 months and whether SweetExposure at 3 to 6 months (10–12 months) predicts SweetLiking at 6 months (12 months).

## Results

### Subjects

Characteristics of the included mother–infant pairs are displayed in Table 1. The infants were on average exclusively breastfed for about 3 months, and more than half of the infants were male (54 %). The majority of mothers (89 %) had a high or medium level of education.

**Table 1.** Mother and infant characteristics, OPALINE* (*n* 319)

Infant characteristics		*n*	%
Sex	Male	172	54
	Female	147	46

		Mean	sd
Weight (kg)	At birth	3·25	0·48
	At 3 months	5·78	0·72
	At 6 months	7·47	0·78
	At 12 months	9·55	0·96
Length (cm)	At birth	49·52	4·55
	At 3 months	60·08	2·74
	At 6 months	66·71	2·52
	At 12 months	74·88	2·66
Duration of exclusive breast-feeding (months)		2·97	1·93
Age at beginning of complementary feeding (months)		5·46	0·84

Maternal characteristics		Mean	sd
Age at delivery (years)		30·75	4·58
Pre-pregnancy BMI (kg/m^2^)		22·14	3·43

		*n*	%
Level of education†	Low	33	11
	Middle	125	39
	High	159	50

* Observatoire des Préférences Alimentaires du Nourrisson et de l’Enfant (Observatory of Infant and Child Food Preferences).

† Level of education: Low = high school diploma or less; middle = secondary education or undergraduate education; high = university or higher vocational training.

### Sweetness liking at 3, 6 and 12 months

**Fig. 1.**
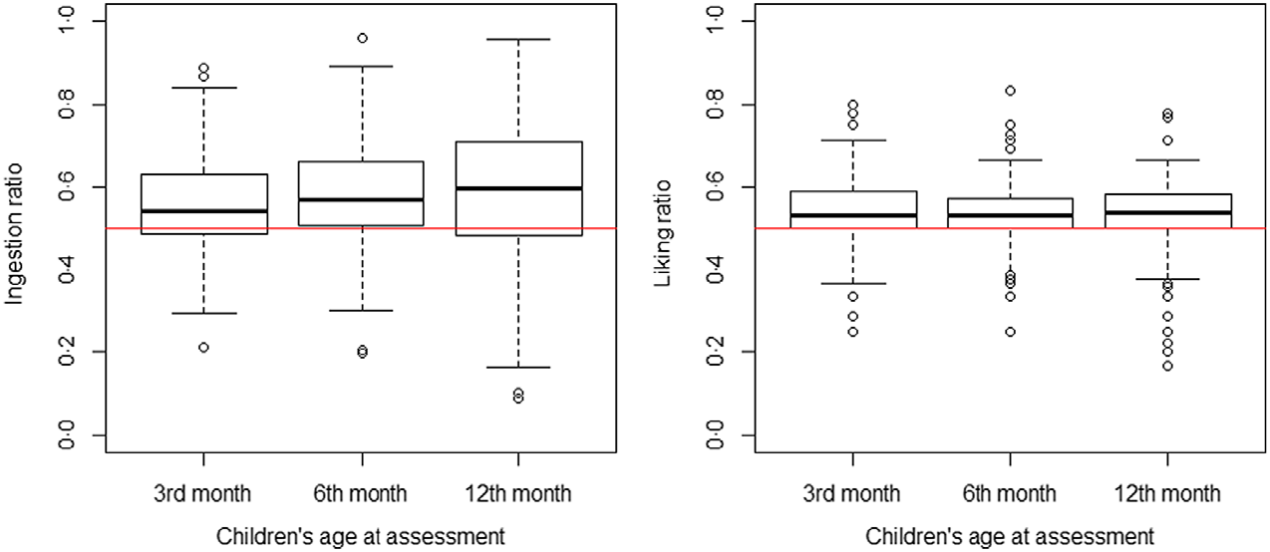
Children’s degree of sweetness liking measured by the ingestion ratio and liking ratio (rated by the experimenter) at 3, 6 and 12 months.

The calculated average IR was 0·56 ± 0·12 at 3 months, 0·58 ± 0·13 at 6 months and 0·58 ± 0·18 at 12 months of age. The calculated average LRE was 0·53 ± 0·09 at 3 months, 0·54 ± 0·08 at 6 months and 0·52 ± 0·10 at 12 months of age (Fig. 1). Hence, on average, the sweet solution was preferred over water at all time points as all the IR and LRE values were above 0·5.

Paired samples *t* tests confirmed that the IR did not change significantly between 3 and 6 months (t(136) = 1·14, *P* = 0·26, CI 95 % (–0·01, 0·05)), between 6 and 12 months (t(163) = 1·14, *P* = 0·26, CI 95 % (–0·01, 0·05)), and neither between 3 and 12 months (t(121) = 1·59, *P* = 0·11, CI 95 % (–0·01, 0·06)). The Levene paired test showed unequal variances with more variability regarding the IR in the twelfth month compared with the third month (t(121) = 2·88, *P* = 0·005, CI 95 % (0·01, 0·06)) or sixth month (t(163) = 3·80, *P* < 0·001, CI 95 % (0·02, 0·06)). However, variances in the IR did not differ between the third and sixth month (t(136) = 0·08, *P* = 0·94, CI 95 % (–0·02, 0·02)). Similarly, paired samples *t* tests confirmed that the LRE did not change significantly between 3 and 6 months (t(136) = −0·57, *P* = 0·57, CI 95 % (–0·02, 0·01)), between 6 and 12 months (t(163) = −0·82, *P* = 0·41, CI 95 % (–0·02, 0·01)), and between 3 and 12 months (t(121) = −0·73, *P* = 0·46, CI 95 % (–0·03, 0·01)). The Levene paired test indicated that the variances did not differ significantly regarding the LRE between the twelfth and third month (t(121) = –1·22, *P* = 0·23, CI 95 % (–0·03, 0·01)), between the twelfth and sixth month (t(163) = 0·98, *P* = 0·33, CI 95 % (–0·01, 0·02)), or between the third and sixth month (t(136) = 1·42, *P* = 0·16, CI 95 % (0·00, 0·02)).

### SweetExposure

**Fig. 2.**
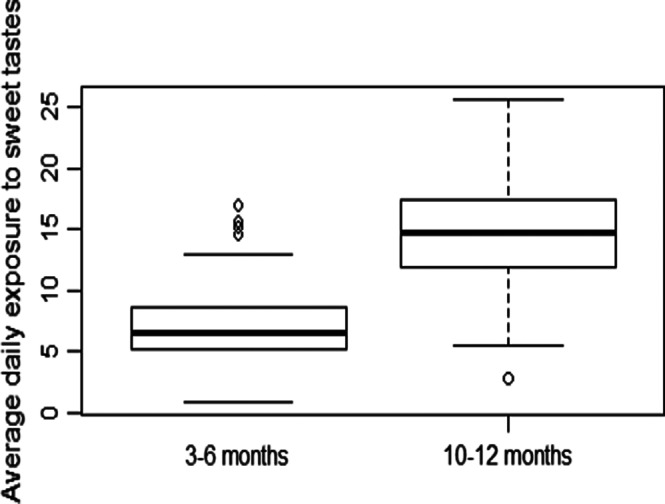
Infants’ exposure to sweetness during the 3 to 6 months and the 10 to 12 months periods.

The daily exposure to sweetness increased significantly from 3 to 6 months to 10 to 12 months as confirmed by the paired samples *t* test (t(246) = 28·64, *P* < 0·001, CI 95 % (7·43, 8·53)). Whereas SweetExposure was on average at 7·07 ± 2·53 during the 3 to 6 months period, it was double as high during the 10 to 12 months period with an average SweetExposure of 14·71 ± 3·87 (Fig. 2). The Levene paired test showed unequal variances with more variability in sweetness exposure in the 10 to 12 months period compared with the 3 to 6 months period (t(246) = 6·16, *P* < 0·001, CI 95 % (0·80, 1·55)).

### Effect of SweetExposure on SweetLiking (regression models)

Neither at 6 months nor at 12 months SweetLiking (indicated by IR and LRE) was associated with prior SweetExposure (Tables 2 and 3). No relationship was found in the bivariate analyses between SweetLiking at 6 months and SweetExposure at 3 to 6 months of age. This was true for both parameters measuring SweetLiking: IR (*β* (95 % CI): 0·006 (–0·002, 0·013)) and LRE (*β* (95 % CI): –0·002 (–0·007, 0·003)). This finding was supported when controlling for the duration of exclusive breast-feeding and the mothers’ educational level, as well as when only including individuals for whom we had complete data on all variables (SweetExposure 3 to 6 months, SweetExposure 10 to 12 months, SweetLiking 6 months and SweetLiking 12 months; Table 2). Moreover, the association between SweetExposure at 10–12 months and SweetLiking at 12 months was not significant: neither for the IR (*β* (95 % CI): –0·002 (–0·008, 0·005)) nor for the LRE (*β* (95 % CI): –0·002 (–0·006, 0·001)). Again, this finding was supported when controlling for the duration of exclusive breast-feeding and the mothers’ educational level, as well as when only including individuals for whom we had complete data on SweetExposure and SweetLiking at 3 to 6 and 10 to 12 months (Table 3).

**Table 2.** Regression models investigating the influence of sweetness exposure at 3 to 6 months on the degree of sweetness liking at 6 months for two sweetness liking outcome variables: ingestion ratio and liking ratio (rated by the experimenter)

		Ingestion ratio					Liking ratio experimenter				
	*n*	*β*	se *	CI 95 %	RSD†	*P*	*β*	se *	CI 95 %	RSD†	*P*
Model 1	182	0·006	0·004	–0·002, 0·013	0·128	0·14	–0·002	0·002	–0·007, 0·003	0·083	0·40
Model 2	158	0·003	0·004	–0·005, 0·011	0·129	0·41	–0·004	0·003	–0·009, 0·002	0·086	0·18
Model 3	150	0·006	0·004	–0·002, 0·015	0·129	0·13	–0·001	0·003	–0·005, 0·005	0·079	0·96

Model 1: Bivariate model (outcome variable: SweetLiking at 6 months (ingestion ratio or liking ratio experimenter); independent variable: SweetExposure at 3 to 6 months), *n* 182.

Model 2: Multiple linear regression model (outcome variable: SweetLiking at 6 months (ingestion ratio or liking ratio experimenter); independent variables: SweetExposure at 3 to 6 months + duration of exclusive breast-feeding + maternal education level), *n* 158.

Model 3: Bivariate model on complete cases (only individuals with complete information about exposure and liking at 3 to 6 and 10 to 12 months) (outcome variable: SweetLiking at 6 months (ingestion ratio or liking ratio experimenter); independent variable: SweetExposure at 3 to 6 months), *n* 150.

* Standard error.

† Residual standard error.

**Table 3.** Regression models investigating the influence of sweetness exposure at 10–12 months on the degree of sweetness liking at 12 months for two sweetness liking outcome variables: ingestion ratio and liking ratio (rated by the experimenter)

		Ingestion ratio					Liking ratio experimenter				
	*n*	*β*	se *	CI 95 %	RSD†	*P*	*β*	se *	CI 95 %	RSD†	*P*
Model 1	197	–0·002	0·003	–0·008, 0·005	0·176	0·61	–0·002	0·002	–0·006, 0·001	0·100	0·23
Model 2	167	–0·002	0·004	–0·009, 0·005	0·178	0·57	–0·003	0·002	–0·007, 0·001	0·102	0·10
Model 3	150	–0·003	0·004	–0·010, 0·004	0·171	0·42	–0·002	0·002	–0·006, 0·001	0·092	0·20

Model 1: Bivariate model (outcome variable: SweetLiking at 12 months (ingestion ratio or liking ratio experimenter); independent variable: SweetExposure at 10–12 months), *n* 197.

Model 2: Multiple linear regression model (outcome variable: SweetLiking at 12 months (ingestion ratio or liking ratio experimenter); independent variables: SweetExposure at 10–12 months + duration of exclusive breast-feeding + maternal education level), *n* 167.

Model 3: Bivariate model on complete cases (only individuals with complete information about exposure and liking at 3 to 6 and 10 to 12 months) (outcome variable: SweetLiking at 12 months (ingestion ratio or liking ratio experimenter); independent variable: SweetExposure at 10–12 months), *n* 150.

* Standard error.

† Residual standard error.

### Structural equation model

**Fig. 3.**
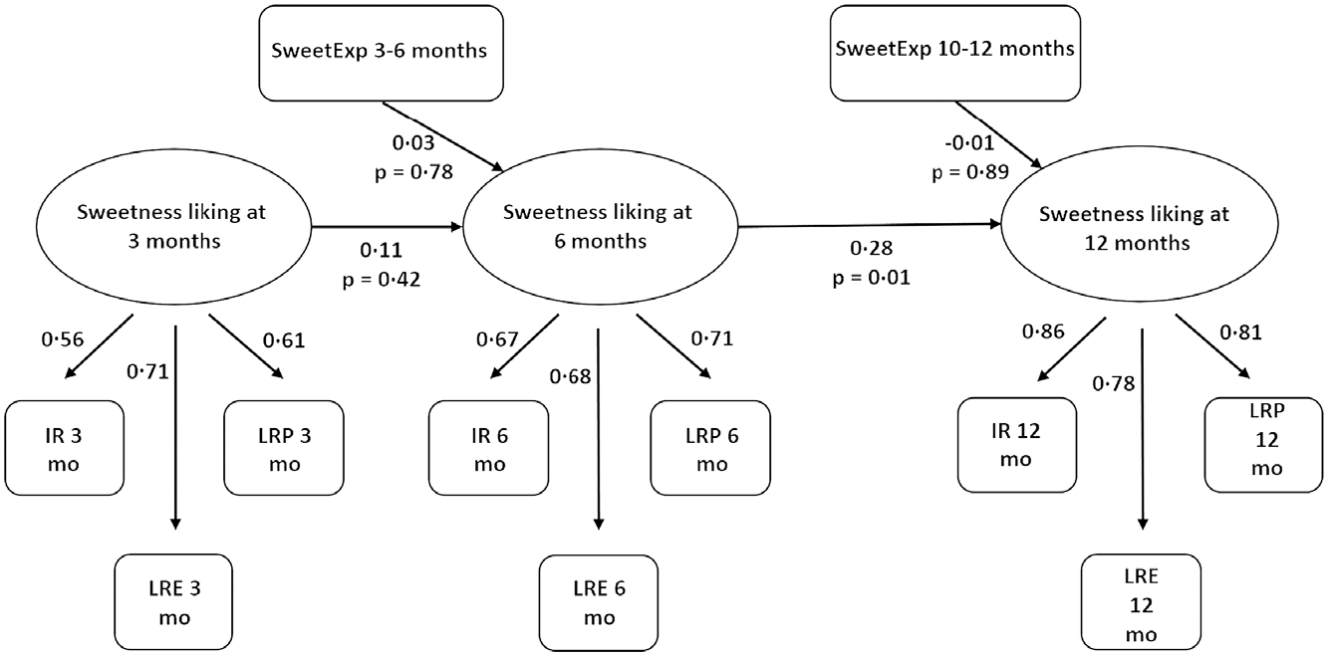
SEM regression model and standardised parameters (*n* 319) showing the relationship between the degree of sweetness liking at 3, 6 and 12 months, as well as the associations between sweetness exposure at 3 to 6 months and the degree of sweetness liking at 6 months; and the relationship between sweetness exposure at 10 to 12 months and the degree of sweetness liking at 12 months. SEM, structural equation model; IR, ingestion ratio; LRE, liking ratio rated by the experimenter; LRP, liking ratio rated by the parent.

The preliminary measurement model showed good fit (CFI = 0·99, TLI = 0·98, RMSEA = 0·03 (95 % CI (0·00, 0·06))), with high loadings (0·56 to 0·86, all *P* < 0·001) and low correlations between latent variables (0·07 to 0·34). The structural model also showed good fit (CFI = 0·97, TLI = 0·96, RMSEA = 0·04) and indicated that SweetLiking at 6 months predicted SweetLiking at 12 months (standardised estimate = 0·28, *P* = 0·01) (Fig. 3). However, SweetLiking at 3 months did not predict SweetLiking at 6 months (standardised estimate = 0·11, *P* = 0·41). In this model, no effect of SweetExposure on SweetLiking was found, as previously concluded from the regression analyses.

## Discussion

To the authors’ knowledge, this is the first study that investigated the relationship between exposure to sweet taste estimated over the whole diet and the degree of sweetness liking in the first year of life, during two major shifts in feeding mode (3 to 6 months – beginning of complementary feeding; 10 to 12 months – transition to the family table). In contrast to our hypothesis, no association between infant’s exposure to sweet taste in the diet and their degree of sweetness liking was found, neither at the beginning of complementary feeding at 6 months nor the transition to the family table at 12 months. Even though, as expected, the exposure to sweet tastes increased from 3 to 6 months to 10 to 12 months, the degree of sweetness liking did not change between these periods. In line with our hypothesis, sweetness liking at 3 months did not predict sweetness liking at 6 months. However, sweetness liking at 6 months did predict sweetness liking at 12 months.

### Sweet taste exposure in infancy

Most birth cohorts focused on nutrition tend to characterise dietary macro- and micro-nutrient intake instead of taste exposure^(39,40)^. Besides other papers investigating the OPALINE cohort, only one recent study focused on taste exposure^(41)^. However, the researchers used a different method than the one used in this study and investigated a slightly older cohort (1 to 2 years) than the age group we focused on. Hence, the level of sweetness exposure in the present study is difficult to compare with other studies for 3 to 12 months old infants. However, to give a reference, the level of sweetness exposure at 3 to 6 months in our study corresponds to a daily average intake of four bottles of infant formula, including one bottle with infant dry sweetened cereals. The level of sweetness exposure at 10–12 months corresponds to three bottles of infant formula, including one with dry sweetened cereals, a vegetable, meat, and starch purée with added fat, two fruit purées, and a fruit-flavoured yogurt^(28)^. In the OPALINE sample, fruits and vegetables were most often introduced first, followed by dairy products, cereal, meats, dessert, starchy foods, fish and biscuits^(30)^. This is comparable with what was found by the French national survey on food consumption of children up until the age of 3 years. Besides milk, the first consumed foods were fruits, vegetables and dairy products, followed by cereals, potatoes, meat, fish, rice and pasta^(42)^.

### Relationship between sweet food consumption and sweetness liking

Previous studies draw contradictory conclusions regarding the link between exposure to sweetness, estimated by the consumption of sweet foods, and sweetness preference in childhood. On the one hand, when measuring sweetness preference using the same sweet food or drink with which sweetness exposure was estimated, the literature suggests a positive relationship between sweetness exposure and liking^(16,17,43)^. This was true for 6-month-old infants who showed a heightened preference for sweetened water after regularly being fed with it during their first 6 months of life compared with children without regular consumption of sweetened water^(16)^. This positive relationship was also found in 5-year-old children who liked a sweetened fruit-flavoured drink more after being exposed to it for 8 d^(43)^. Those findings contradict the present results where the association between sweetness exposure and the degree of sweetness liking over the first year of life could not be proven. This may be explained by the fact that in this study the relationship between sweetness exposure and liking was analysed by looking at the overall dietary exposure to sweet taste in the diet, whereas the papers above looked at the change in sweetness liking for the same sweet beverage the child had been exposed to. Previous studies demonstrated that the association between sweetness exposure and liking is not as straightforward when exposure and liking are tested with different mediums. For example, Beauchamp and Moran^(17)^ could not replicate the positive relationship between sweetness exposure (through sweetened water) at 6 months and liking when testing sweetness liking with a fruit-flavoured drink instead of sweetened water at 2 years. However, when sweetness liking was assessed with sweetened water at this age, the association was still positive^(17)^. Furthermore, a systematic relationship between consuming sweet products and sweetness liking in 7-to-12-year-old children could not be proven, as sweetness liking was only related to candy and snack consumption but not to consumption of sweet drinks, dairy products, fruit, cereal or added sugar consumption^(19)^. This suggests that the association between exposure and liking is largely stimulus-specific and cannot be generalised to the whole diet. As children learn through repeated exposure which foods should taste sweet^(5)^, children may get used to the taste of the specific sweetened beverages which resulted in an increased liking as previously shown^(16,17,43)^. However, this is not associated with a general increase in children’s preference for sweetness. This does not undermine the importance of food and flavour experiences, in particular during the first years of life, in the shaping of food preferences, but emphasises the fact that this learning is food-specific^(22)^.

### No relationship between sweetness exposure and sweetness liking: possible interpretation

We hypothesised that higher exposure to sweet tastes during infants’ first year of life may result in a higher degree of sweetness liking. However, the results did not confirm this hypothesis. Apart from the possibility that this hypothesis is wrong, other aspects must be considered to interpret these findings. First, individual variations in the degree of sweetness liking at this early age may still be a result of genotypes, and not of environmental influences such as dietary exposures. This assumption is supported by a study by Mennella *et al.* ^(44)^ that found that in 5-to-10-year old children, the TAS2R38 taste gene locus was related to sucrose preferences and the liking of sweet-tasting foods and drinks, which was much less obvious in their mothers. Nonetheless, following this line of reasoning, sweetness liking at 3 months should have predicted sweetness liking at 6 months in our sample, yet it did not, even though sweetness was already preferred over water at 3 months. However, sweetness liking at 6 months did predict sweetness liking at 12 months. This result may be due to the fact that infants’ control over swallowing behaviour at 3 months of age was still too limited to reflect individual differences well enough, although it appeared adequate to reflect a preference for the sweetened water over plain water.

Second, sweetness exposure in this study sample may not have been varied enough to detect an effect on the degree of sweetness liking. This may be especially true because the parents in our study started to complementary feed their infants on average at the age of five and a half months, with little variability. Hence, infants may have been exposed to comparable sweetness intensity levels for most of the time in the first period due to exclusive milk feeding. In the second period when sweetness exposure varied more between infants, the duration of the exposure period (between 10 and 12 months) may have been too short, or the sweet taste intensities may not have been strong enough to detect an effect on sweetness liking. Also, Schwartz *et al.* ^(33)^ found that during the first 12 months, the taste intensities of the foods children were exposed to were relatively low, even though children mainly experienced sweet taste during the milk feeding period with an even increased sweetness exposure during the first year. Because in this study only exposure frequency to sweet tastes was assessed, but not how much of the sweet-tasting foods were consumed in each eating occasion, we cannot estimate the proportion of the overall energy intake coming from sweet-tasting foods. Hence, it must be considered that the overall sweetness intensity the infant was exposed to in the first 12 months of life was not strong enough to detect an effect on sweetness liking.

Third, the effect of sweetness exposure in infancy may affect the degree of sweetness liking only later in childhood, after a longer period of exposure to sweet-tasting foods. Liem and Mennella^(14)^ found that children from parents who regularly added sugar to the child’s diet between 0 and 12 months were more likely to prefer apple juice with added sugar at 4 to 7 years. In line with this result, a diet rich in added sugars during the first 12 months was related to a higher degree of sweetness liking at 3 to 7 years^(13)^. In the present study, the IR during the sweetness liking test in the twelfth month varied more between children than in the third and sixth months. In combination with the increased exposure to sweetness in the second period (10–12 months), this may hint towards the possibility of a slight impact of sweetness exposure on the degree of sweetness liking, although this link was not detected in our analyses.

Lastly, growing evidence demonstrates that hedonic responses to sweet taste differ between individuals due to sweet-liking phenotypes^(12)^. It was found that children can be classified as either sweet likers or sweet dislikers^(6)^. Hence, children in our sample may also move along this continuum. The unequal variances regarding the IR may be a hint towards those differences in sweetness liking in our sample. However, we expected that sweetness exposure would lead to a similar effect across the continuum of children who rather like or dislike sweetness. Nevertheless, repeated exposure to sweetness could have led to a different effect on infants depending on their sweet-liking phenotype and may have influenced our outcome measure.

### Strengths, limitations and conclusion

Despite the care which was taken to account for uncontrolled factors, our study entails some limitations. First, in our study, only consumption frequencies were recorded, not portion sizes. Hence, infants with the same exposure frequencies of sweet-tasting foods but different portion sizes were assigned the same level of sweetness exposure. Thus, we cannot account for the contribution of portion size to sweetness exposure. Further, the methodological choice to measure sweetness liking may have undermined the liking value, because some infants may have not been used to the taste of sweetened water, since they were mostly fed milk during the first months of life. However, lactose was chosen to create the sweetened water solution as it is a taste infants were likely to have experienced, in particular in breast, or formula milk^(31)^. In addition to that, sweetness exposure was estimated based on the food records mothers filled out. We cannot assure that mothers have filled in the food records truthfully. Further, when a sweet food (e.g. applesauce) was consumed with sour food (e.g. yogurt), the sourness of the sour food may have reduced the perceived sweetness of the sweet food. Hence, we cannot judge the infants’ perceived sweetness exposure experience, but only sweetness exposure based on the rated sweetness intensity levels. Lastly, similar to other cohort studies, lower educated families were under-represented in our sample as almost 90 % of mothers had a high or medium level of education. A higher maternal education level is generally associated with better diet quality in mothers and children and was previously associated with higher exposure to neutral tastes in children aged 1 and 2 years^(41,45,46)^. Following that, conclusions from our study are less applicable to children of lower-educated mothers. It has to be mentioned that the subjective sweetness liking experience cannot be truly measured, but only relative sweetness liking. Following that, in our study, the degree of sweetness liking refers to a relative liking of sweetness compared with neutral stimuli (water). However, a strength of our study is that three proxies for relative liking were included in our analyses (IR, LRE and LRP) to get a broader picture of relative sweetness liking. Besides the limitations, our study contains multiple strengths. First, infants’ overall exposure to sweetness was taken into account instead of focusing on the macro-nutrient intake only. For that, the overall diet was included and not only exposure to particular sweet-tasting foods. Another strength is the longitudinal design which allowed examination of sweetness exposure and liking at multiple time points. Further, the fact that sweetness exposure was estimated based on twelve monthly 7-d food records gave an overall impression of infants’ exposure to sweetness over the first year instead of only delivering a snapshot of sweetness exposure at a single time point. As sweetness exposure was averaged based on the monthly food records for each of the periods, the impact of outliers (e.g. due to special occasions such as birthdays) regarding the intake of sweet-tasting foods was minimised.

In conclusion, this study did not show a relationship between sweetness exposure and the degree of sweetness liking in infants throughout observations from the third until the twelfth month of life. Although sweetness exposure increased from the first (3 to 6 months) to the second period (10 to 12 months), the degree of sweetness liking did not change. This was contrary to what was expected. Based on the findings, we hypothesise that, at such early age, inter-individual variations in sweetness liking may rather occur due to differences in genotypes, especially when variations in sweetness exposure between children are limited. The impact of high exposure to sweet-tasting foods in the first 12 months of life on the degree of sweetness liking later in life should be investigated longitudinally including time points in childhood up until adolescence to understand the impact of sweetness exposure on establishing (healthy) eating habits early on. Longitudinal research has shown that eating behaviour learned in infancy impacts eating behaviour in childhood^(47)^. Whether this is also true for early exposure to sweet taste remains unclear. Future studies should aim for more variability in the study sample with participants representing the whole sociodemographic continuum, to represent a variety of feeding habits, and should also consider the consumed quantities of sweet products in addition to the frequencies.

## Supplementary material

For supplementary material accompanying this paper visit https://doi.org/10.1017/S0007114522002628. click here to view supplementary material
