# Supplementary material for: The sweet tooth of infancy: Is sweetness exposure related to sweetness liking in infants up to 12 months of age?
Source: Br J Nutr. 2022 Aug 11;129(8):1462–72. doi: 10.1017/S0007114522002628 (PMC10024975; doi:10.1017/S0007114522002628)
Supplement: Supplementary file 1 [file S0007114522002628sup001.docx]

**Supplementary Material:**


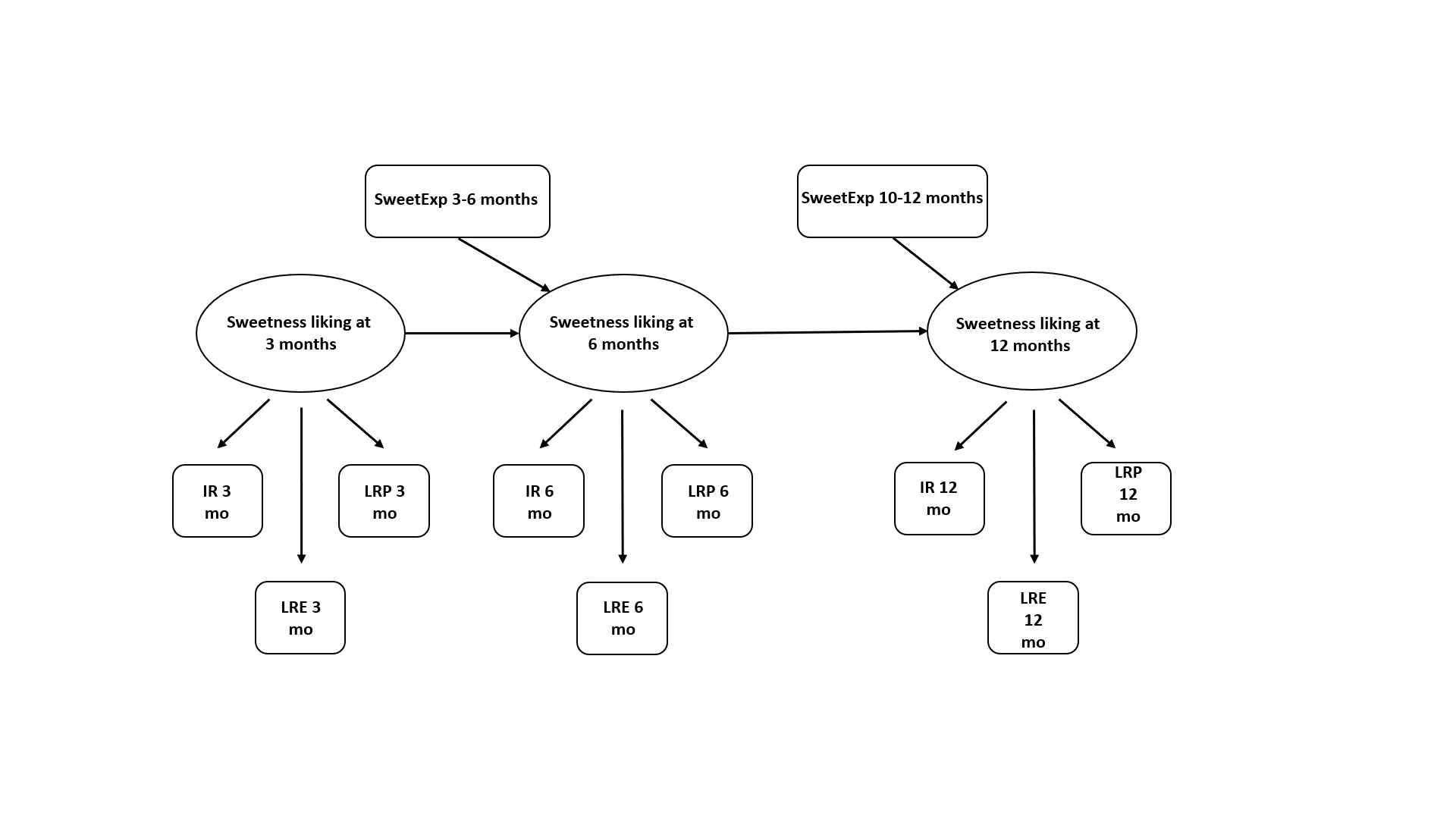


**Figure S1.** SEM model structure showing the relationship between sweetness liking at three, six, and twelve months, as well as the associations between sweetness exposure at three to six months and sweetness liking at six months; and the relationship between sweetness exposure at ten to twelve months and sweetness liking at twelve months. IR: Ingestion ratio; LRE: Liking ratio rated by the experimenter; LRP: Liking ratio rated by the parent.
